# Predictors of Left Ventricular Mass Regression Following Aortic Valve Replacement

**DOI:** 10.1186/1749-8090-10-S1-A86

**Published:** 2015-12-16

**Authors:** Farouk M Oueida, Ibrahim M Yassin, Mostafa A Eissa

**Affiliations:** 1 Cardiac Surgery Department, Saud al-Babtin Cardiac Center (SBCC), Al-Dammam, 31463, KSA; 2 Cardiac Surgery Department, Tanta University Hospitals, Tanta, 31111, Egypt

## Background/Introduction

Aortic stenosis (AS) is one of the most common valvular heart disease nowadays Independent factors affecting the postoperative outcome had been studied long time ago.

## Aims/Objectives

Identification of the predictors of left ventricular mass regression after aortic valve replacement is our aim of this study.

## Method

Randomized selection of 100 patients, underwent aortic valve replacement with a single type of bio-prosthesis (Medtronic Mosaic) for pure aortic stenosis. The study population showed that, 25/100 (25%) patients had prosthesis-patient mismatch of a moderate degree (indexed effective orifice area (IEOA) from 0.65 cm2/m2 - 0.85 cm2/m2). The effect of prosthesis-patient mismatch on the postoperative echocardiographic findings mainly the regression of left ventricular mass after aortic valve replacement and follow up comparison of the unmatched group with the matched group in addition to the other possible related factors through the multivariate analysis was studied.

## Results

In multivariate analysis, hypertensive patients, preoperative New York Heart Association (NYHA) class >II and a higher preoperative left ventricular mass ≤250 g/m2 are independent predictors of incomplete left ventricular mass regression. Age and Gender was found to be insignificant predictors. There was a good correlation (r = 0.755, p < 0.001) between the postoperative left ventricular mass regression (LVMR) and the projected indexed effective orifice area. There was a significant reduction of left ventricle (LV) mass in both groups and a significant reduction of LV mass index among Non PPM group while it was of a no significant reduction in PPM.

## Discussion/Conclusion

This study shows that in patients with pure aortic stenosis prosthesis-patient mismatch is associated with lesser regression of left ventricular hypertrophy after aortic valve replacement. Hypertension, preoperative (NYHA) class >II and a left ventricular mass ≤250g/m2 are other independent predictors.

